# Investigating intra-host and intra-herd sequence diversity of foot-and-mouth disease virus^☆^

**DOI:** 10.1016/j.meegid.2016.07.010

**Published:** 2016-10

**Authors:** David J. King, Graham L. Freimanis, Richard J. Orton, Ryan A. Waters, Daniel T. Haydon, Donald P. King

**Affiliations:** a The Pirbright Institute, Ash Road, Pirbright, Woking, Surrey GU24 0NF, UK; b Institute of Biodiversity, Animal Health and Comparative Medicine, College of Medical, Veterinary and Life Sciences, University of Glasgow, Glasgow, G12 8QQ, UK; c MRC-University of Glasgow, Centre for Virus Research, University of Glasgow, 464 Bearsden Road, G61 1QH, UK

**Keywords:** Foot-and-mouth disease, Next generation sequencing, Variant analysis, Phylogenetics, Viral diversity

## Abstract

Due to the poor-fidelity of the enzymes involved in RNA genome replication, foot-and-mouth disease (FMD) virus samples comprise of unique polymorphic populations. In this study, deep sequencing was utilised to characterise the diversity of FMD virus (FMDV) populations in 6 infected cattle present on a single farm during the series of outbreaks in the UK in 2007. A novel RT–PCR method was developed to amplify a 7.6 kb nucleotide fragment encompassing the polyprotein coding region of the FMDV genome. Illumina sequencing of each sample identified the fine polymorphic structures at each nucleotide position, from consensus level changes to variants present at a 0.24% frequency. These data were used to investigate population dynamics of FMDV at both herd and host levels, evaluate the impact of host on the viral swarm structure and to identify transmission links with viruses recovered from other farms in the same series of outbreaks. In 7 samples, from 6 different animals, a total of 5 consensus level variants were identified, in addition to 104 sub-consensus variants of which 22 were shared between 2 or more animals. Further analysis revealed differences in swarm structures from samples derived from the same animal suggesting the presence of distinct viral populations evolving independently at different lesion sites within the same infected animal.

## Highlights

• NGS was used to characterise FMD viruses in clinical samples from cattle.
• 5 consensus and 104 sub-consensus level substitutions were identified.
• Distinct virus swarms were found in different lesions within the same host.
• Only 22 sub-consensus substitutions were shared between 2 or more animals.
• Data suggest that FMDV evolves independently at different lesion sites.

## 1 Introduction

Foot-and-mouth disease virus (FMDV) causes an economically devastating vesicular disease of domesticated and wild cloven hoofed animals and is considered the single most important constraint to the international live-stock trade (Perry and Rich, 2007) as incursions into disease-free countries result in significant economic and trade restrictions (Pendell et al., 2007). FMDV is a positive sense single stranded RNA virus belonging to the genus *Aphthovirus* within the Picornaviridae family. The genome of approximately 8.3 kilobases in length contains a single open reading frame (with two alternative start codons), which encodes for 4 structural proteins (VP1–VP4) and 11 non-structural proteins (leader [L_ab_, L_b_], 2A–2C, 3A, 3B_1_, 3B_2_ and 3B_3_, 3C and 3D) and is flanked by two untranslated regions (UTR) (Yoon et al., 2011).

RNA viruses such as FMDV exist within a host as a heterogeneous population (Haydon et al., 2001). This genetic diversity occurs due to the poor proof-reading ability of the viral RNA dependent RNA polymerase, together with the large viral population size and the high replication rate (Yoon et al., 2011). From current RNA polymerase error rates estimates [between 10^− 3^ and 10^− 6^ substitutions per nucleotide per replication event (Drake, 1993, Sanjuán, 2010, Thébaud et al., 2010)], it can be hypothesised that at least one nucleotide change occurs in each FMDV genome per transcription event (Haydon et al., 2001).

Re-construction of viral transmission pathways using molecular sequence data is an important component of foot-and-mouth disease (FMD) control strategies, which relies on phylogenetic analysis of viral sequences recovered from field cases (Knowles and Samuel, 2003). These methods typically use genetic data generated from whole genome or a single coding region e.g. viral protein-1 (VP1) sequencing. Due to its genetic diversity, the VP1 coding region is widely used for global tracing of FMDV (Knowles and Samuel, 2003), however over shorter epidemic time scales such a short fragment may not be able to discriminate between closely related viruses. Therefore, the increased resolution afforded by whole genome sequencing is a powerful tool for reconstructing fine-scale transmission pathways (Cottam et al., 2008, Wright et al., 2013).

While consensus-level sequencing of FMDV is relatively commonplace in molecular epidemiology, the sub-consensus diversity remains largely uncharacterised. The resolution afforded by consensus sequencing will not always resolve the fine scale processes that drive viral evolution, with the importance of minor variants remaining unclear in relation to both FMDV transmission and evolution (Holmes and Moya, 2002). Although the possibility remains of using cloning and Sanger sequencing to identify low frequency genomic changes, this procedure is both expensive and resource-intensive (Cottam et al., 2009). Bench top next generation sequencing (NGS) technologies such as Illumina's MiSeq and Life Technologies' PGM have provided efficient and increasingly affordable ways to ‘deep sequence’ viral population diversity (Wright et al., 2011, Logan et al., 2014). NGS platforms have already been applied to FMDV, to generate both consensus and sub-consensus level sequences (Morelli et al., 2013, Logan et al., 2014, Wright et al., 2011). These studies highlight the ability of NGS technologies to facilitate the analysis of FMDV evolution, at a high-throughput and high resolution scale. Processed-introduced changes during the sample preparation steps and errors during base-calling confounds the identification of true low frequency viral variants, although there are now computational methods that aim to distinguish true viral variants from erroneous substitutions (Yang et al., 2013, Wilm et al., 2012, Orton et al., 2015).

In this study, Illumina sequencing was used to produce a snapshot of genetic diversity of the FMDV polyprotein coding region, at both intra-host and intra-herd levels. In total 7 samples were collected from 6 animals, from a single FMDV infected premises (IPs). IP2b was part of a larger series of outbreaks that comprised 8 infected farms, constituting 11 holdings, in the south-east of England in 2007, the causative agent of which was identified as FMDV O_1_ British field sample 1860 (O_1_BFS 1860) (Cottam et al., 2008). Although a number of viral samples from these farms have already been characterised at the consensus level (Cottam et al., 2008, Valdazo-González et al., 2015), the underlying viral populations at the sub-consensus level remain unexplored, along with important questions regarding intra-host and intra-herd diversity and transmission bottlenecks.

## 2 Materials and methodology

### 2.1 Viral specimens

The protocol for RT and PCR were optimised and validated using a single FMDV field isolate - FMDV O_1_BFS 1860/UK/67 (NCBI accession number EU448369), which was cultured in primary bovine thyroid cells (BTY), as described previously (Snowdon, 1966). The optimised RT–PCR protocol was subsequently applied to 7 bovine epithelial samples collected from 6 FMDV positive cows, which were part of a beef sucker herd on a single infected premise (IP2b) during the series of FMD outbreaks that affected the United Kingdom (UK) in 2007 (Ryan et al., 2008). Samples were labelled according to the last 3 digits of the infected animal's tag identification number, with 2 samples deriving from the same animal but different lesions labelled as sample 161A and sample 161B. Data such as estimated lesion age was taken from original veterinary records recorded during the outbreaks (Table 1).

### 2.2 Sample preparation

A 10% original suspension was made from bovine epithelial tissue using M25 phosphate buffer (35 mM Na_2_HPO_4_ 5.7 mM KH_2_PO_4_; pH 7.6). Of this, 460 μl was processed with the RNeasy mini kit (Qiagen, Manchester, UK), according to manufacturer's instructions and eluted in 50 μl of nuclease free water. Total RNA was quantified using the Qubit RNA BR assay (Life Technologies, Paisley, UK) and viral RNA was quantified using a FMDV-specific qRT–PCR assay as described previously (Callahan et al., 2002) with a standard RNA generated from the 3D region of FMDV isolate UKG/35/2001. Prior to further processing, the quantity of FMD viral RNA in the samples was normalised to one another by diluting in FMDV-negative RNA (extracted from bovine epithelial tissue) to 1 × 10^5^ viral copies per μl (cp/μl).

### 2.3 First-strand cDNA synthesis

Reverse transcription was performed using Superscript III reverse transcriptase (Life Technologies, Paisley, UK) as per the manufacturer's instructions.

Briefly reverse transcription was carried out using 14 μl of total RNA, 2 μM of Rev6 [an oligo-T primer (Logan et al., 2014)], 10 mM of dNTPs, 10 × reaction buffer, 25 mM MgCl_2_, 40 units (U) of RNaseOut, 0.1 M DTT and 200 U of Superscript III. The reaction was incubated at 50 °C for 50 min prior to an addition of 2 U of RNase H and a final incubation step of 37 °C for 30 min.

### 2.4 PCR amplification

Primers were designed to amplify a 7.6 kilobase (kb) fragment of the FMDV O_1_BFS 1860 genome, which started within the 5′UTR and ended after the stop codon of the polyprotein encoding region. PCR reactions were performed in duplicates using KAPA high fidelity Taq polymerase enzyme (KAPA Biosystems, London, UK); each PCR duplicates was subsequently sequenced in order to allow reliable identification of low frequency variants in the viral population.

Briefly, 25 μl of 2 × KAPA HiFi HotStart DNA Polymerase ready mix (containing 0.5 U of KAPA HiFi HotStart DNA Polymerase, 0.3 mM of each dNTP and 2.5 mM of MgCl_2_) was added with 1 μM of Forward primer [O_1_BFS 517F (ACGACAAACACGCACAGTTT), genome location 517–536] and 0.3 μM of Reverse primer [O_1_BFS 8159R (TAAGGAAGCGGGAAAAGCCC), genome location 8140–8159] to 3 μl of cDNA. Cycling conditions were; 98 °C for 3 min followed by 35 cycles of 95 °C for 30 s, 65 °C for 15 s, 72 °C for 4 min with a final cycling step of 72 °C for 8 min.

A 1% agarose gel (Sigma-Aldrich, Poole, UK) was used to visualise the 7630 bp PCR products which were then excised and purified using the GFX column purification kit (GE Healthcare, Chalfont, UK). Finally, DNA was quantified using the Qubit® dsDNA High Sensitivity Assay Kit (Life Technologies, Paisley, UK) and diluted to 0.2 ng/μl in nuclease-free water prior to sequencing library preparation.

### 2.5 Illumina sequencing

DNA libraries were constructed using the Nextera XT DNA sample preparation kit (Illumina, San Diego, USA). Final libraries were multiplexed and diluted to 12.5 pM prior to sequencing on a MiSeq (Illumina, San Diego, USA), on a single 2 × 150 cycles, paired-end sequencing run using a version 2 MiSeq reagent cartridge. Sequencing libraries were constructed for all PCR duplicates in order to allow reliable identification of low frequency variants in the viral population.

### 2.6 Data filtering and analysis

Quality control checks were performed on the raw FASTQ data using FastQC (version 0.11.2) (Andrews, 2010). Reads below a quality score of q30 and under a length of 50 bp were removed using Sickle (version 1.33) (Joshi and Fass, 2011). BWA-MEM (version 0.7.12) (Li, 2013) was used to align the reads to a previously determined full genome sequence from IP1b (NCBI accession number EU448372) (Cottam et al., 2008). BAM formatted files were generated using SAMTools (version 0.1.19) (Li et al., 2009), alignments were visually checked using a Tablet (version 1.14.10.20) (Milne et al., 2013) and coverage data and graphs were generated using BEDTools (version 2.18) (Quinlan and Hall, 2010). Finally consensus sequences were constructed using DiversiTools (Hughes and Orton, 2016).

Multiple sequence alignments and consensus sequences were performed in Bioedit (version 7.2.5), alignments were formatted to Phylip format using DNAsp (version 5.10.1) and statistical parsimony analysis was conducted in TCS (version 1.21) (Clement et al., 2000) with previously generated consensus sequences from IP2B (Valdazo-González et al., 2015, Cottam et al., 2008) to produce a TCS network.

### 2.7 Variant analysis

Lofreq (Wilm et al., 2012) was used to identify variants at both consensus and sub-consensus levels using the recommended settings for RNA viruses (holmbonf strand bias filter and non-incorporation of mapping qualities). As sample amplification and NGS sequencing are error prone processes, each sample was PCR amplified and sequenced in duplicate, and only variants called by Lofreq in both replicates were considered valid. The validated LoFreq variant population identified within each sample was then compared to identify the number of variants shared between each of the samples. Shared variants were visualized using the Circos software package (Connors et al., 2009).

## 3 Results

### 3.1 RNA extraction and library complexity

**Fig. 1:**
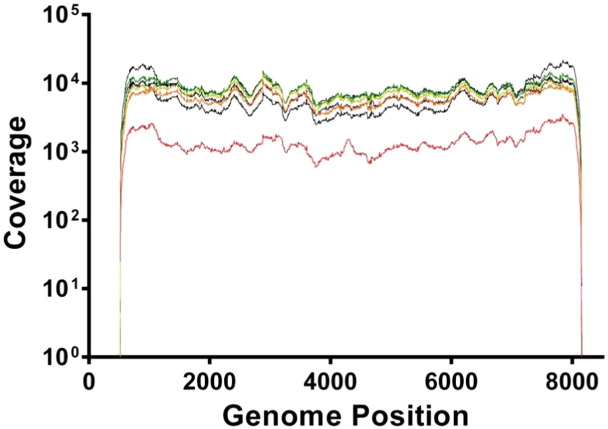
The average coverage distribution for the sequenced samples. For both PCR duplicate sets (with filtered, trimmed reads), average coverage values were between 5.79 × 10^2^ and 1.00 × 10^4^ nucleotides/site, with the mean of all samples being 6.44 × 10^3^ nucleotides/site. Each sample is indicated by a different colour. Sample 147 – red; sample 161A – orange; sample 004 – yellow; sample 241 – green; sample 161B – dark blue; sample 341 – black, and sample 238 – grey.

FMDV quantified by qRT–PCR in samples ranged between 1.19 × 10^5^ (sample 341) and 2.50 × 10^7^ (sample 161b) viral copies/μl (median of 8.51 × 10^6^). Comparison of the qRT–PCR results with the estimated lesion age revealed an inverse correlation, with samples with a lesion age of 6 days having an average of 3.03 × 10^5^ viral copies/μl and samples with a lesion age of 2 or 3 days having an average of 1.35 × 10^7^ viral copies/μl (Table 1). The MiSeq produced a total of 3.18 × 10^6^ paired end reads for all samples, with an average of 4.55 × 10^5^ paired end reads generated for each duplicated sample. Approximately 14% of these reads were eliminated by quality filtering. Of the remaining reads, between 78% (sample 147) and 99.99% (sample 341) mapped to the FMDV O_1_BFS reference genome (mean = 96%). The average coverage depth at each genome position across the amplified fragment ranged from 5.79 × 10^2^ to 1.00 × 10^4^ reads (mean = 6.44 × 10^3^) (Table 1; Fig. 1).

### 3.2 Consensus genetic diversity

Consensus level sequences of amplicons were produced in duplicate, with all replicates exhibiting identical sequences, sequences derived from separate lesions from the same animal (161A and 161B) were also identical. In total, 5 consensus level substitutions were observed across the amplified region of the genome, 2 substitutions were located within the VP2 coding region, with 1 found in the 5′UTR, 2C and 3D coding regions respectively (Table 1). Within the polyprotein coding region, 2 of these substitutions were synonymous and 2 were non-synonymous. Two of these changes within the VP2 (genome position 2429) and 2C coding regions was also present as minor variants in sample 147 (12.18% and 2.53% respectfully).

**Fig. 2:**
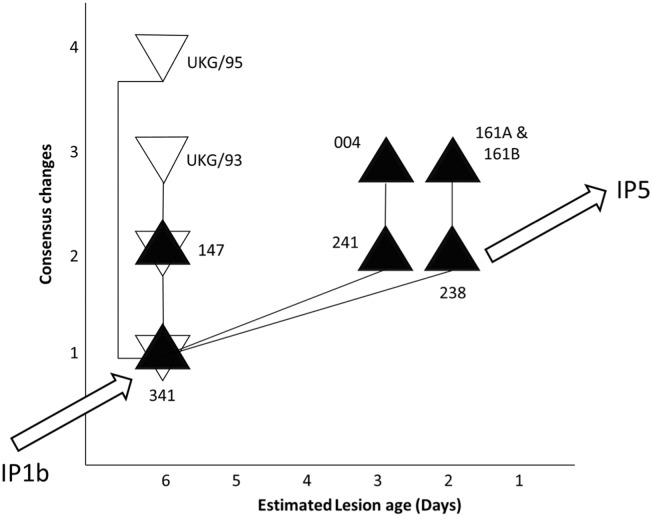
Statistical parsimony analysis of consensus sequences generated in relation to the estimated lesion age of each sample. Grey triangles represent samples that were sequenced as part of this study, white triangles (i.e. UKG/95 and UKG/93) indicate samples from IP2b sequenced previously by Sanger methods (Valdazo-González et al., 2015). Black lines represent single nucleotide substitutions. Closest consensus sequences for viruses recovered from other farms infected in sequence (IP1b and IP5) are indicated.

Statistical parsimony analysis was used to generate a transmission network to investigate the genetic relatedness between FMDV sequences recovered on IP2b (Fig. 2). The consensus sequence of Sample 341 was most genetically similar to FMD viruses collected from IP1b (1 nucleotide difference) and IP2c (2 nucleotide differences) (data not shown in figure). Sample 341 and 147 (both 6 days old) were identical to previously published consensus sequences derived from the same animals on IP2b (GenBank accession numbers: KJ560280 and KJ560282 respectively). Sample 238 exhibited a single nucleotide difference to sample 341, but also demonstrated close nucleotide identify to the consensus sequences derived from IP5 and later IPs (Fig. 2). Over the amplicon region compared in this study, the consensus sequence of sample 004 was also found to be identical to a previously published sequence from IP2b (EU448373; data not shown); however original records at The Pirbright Institute show that these sequences derive from different animals.

### 3.3 Sub-consensus genetic diversity

**Fig. 3:**
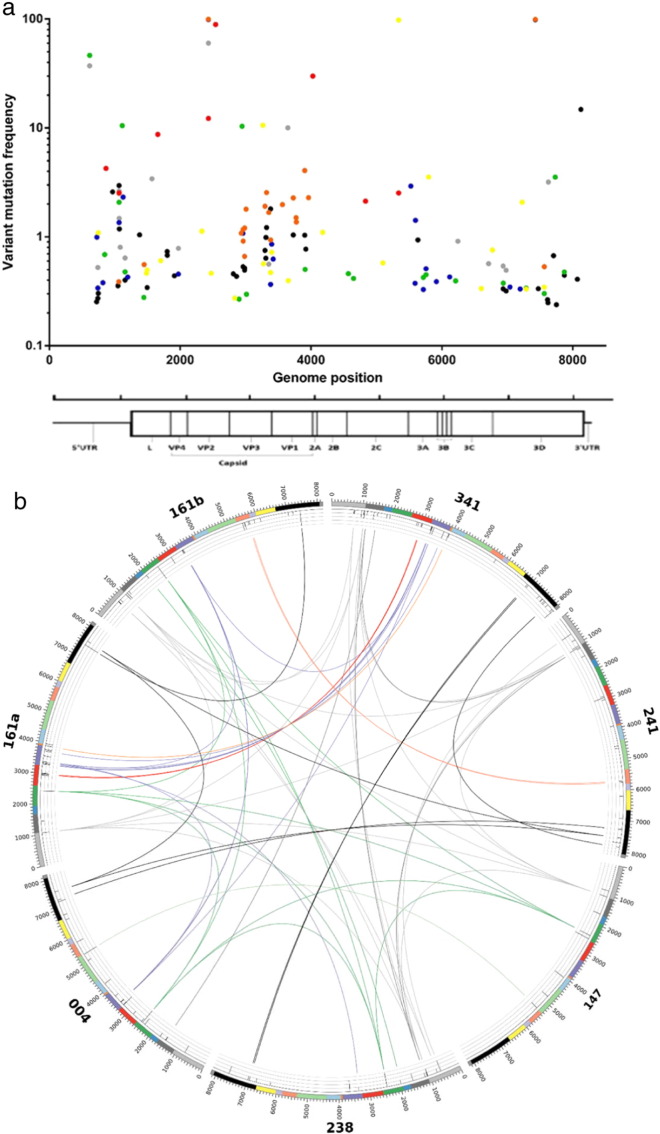
a: The distribution of variants across the FMDV genome. The variants belonging to each sample are indicated by a difference in colour. Sample 147 – red; sample 161A – orange; sample 004 – yellow; sample 241 – green; sample 161B – dark blue; sample 341 – black, and sample 238 – grey. b: A Circos histogram showing the relationship between FMDV consensus and sub-consensus variants. Only variants that were called by Lofreq in both duplicates and shared between 2 of more samples are represented here. The histogram is split into 7 sections, with each representing a different sample. Each genome region is represented by a different colour (light grey – 5′UTR, dark grey – leader, dark blue – VP4, dark green – VP2, red – VP3, dark purple – VP1, dark orange – 2A, light blue – 2B, light green – 2C, light orange – 3A, light purple – 3B, yellow – 3C, black – 3D, grey – 3′UTR). Variants which are shared between 2 or more samples are indicated with links, with the colour correlating to the coding region the variant is present in. The inner circles represent the variant frequencies across the genome. In pseudo log scale, the first gridline represents variants from 0% to 1%, the second gridline represents variants from 1% to 10%, the third gridline represents variants from 10% to 50% and the fourth gridline represents variants from 50% to 100%. For visual purposes, variants were scaled up, 0% to 1% was scaled up to 1%, 1% to 10% was scaled up to 10%, 10% to 50% was scaled up to 50% and 50% to 100% was scaled up to 100%.

The majority of the diversity characterised by Lofreq in all samples was sub-consensus, with 104 unique variants identified with a frequency between 0.24% and 50%. Overall, the mean number of variants for each sample was 19.7, with sample 341 having the highest number (37) and sample 147 having the lowest number (7). The total number and frequency of variants found within each coding region can be found in Table 2 and in Fig. 3a. For this sample set, the highest and lowest relative mutational frequencies were observed in the sequences that encode 2A and 2C, respectively; however, analyses of more samples may be required to properly determine whether these fluctuations across the genome are reproducible and reflect an aspect of FMDV replication and transmission.

Eighty-eight variants were observed in the polyprotein coding region. Of these, 45 were synonymous and 43 were non-synonymous substitutions, with the VP1 coding region containing the highest number of synonymous changes (total of 12) and the 3D coding region containing the highest number of non-synonymous changes (total of 16). To correct for the differences in length, the relative frequencies of variants within each genomic region were calculated, with the 2A coding region having the highest frequency of variants (5.55%) and the 2C coding region having the lowest frequency (0.21%) (mean of 1.64%, Table 2). In total, 37 sub-consensus variants were identified within the capsid coding regions, of which 24 were synonymous and 13 were non-synonymous (Table 2).

Variants identified in IP2b were compared against consensus sequences reported previously for later IPs in the outbreak, in order to ascertain whether sub-consensus diversity in virus populations early on in an outbreak were predictive of future consensus sequences observed on farms that were infected at later time points. Minority variants on IP2b were identified to become fixed at consensus level in later IPs, at 3 locations; 5′UTR (base position: 1082) appearing at IP5, VP2 (base position: 2429) appearing in IPs 3, 4, 5, 6 and 7, and VP3 (base position: 3650) appearing later in IP4b.

The prevalence of sub-consensus variants shared between multiple animals maybe indicative of selective pressures and was investigated further, with a total of 22 sub-consensus variants shared between 2 or more animals. A single variant located within the 5′UTR was shared between 6 samples (mean frequency: 2.34%). The number of shared variants amongst 4 samples, 3 samples and two samples was 1, 3 and 17 respectively (Fig. 3b), suggesting that the majority of shared variants were only present in small numbers of animals. A high proportion of these shared variants were either synonymous (54.54% in total) or in the 5′UTR (9.09% in total).

**Fig. 4:**
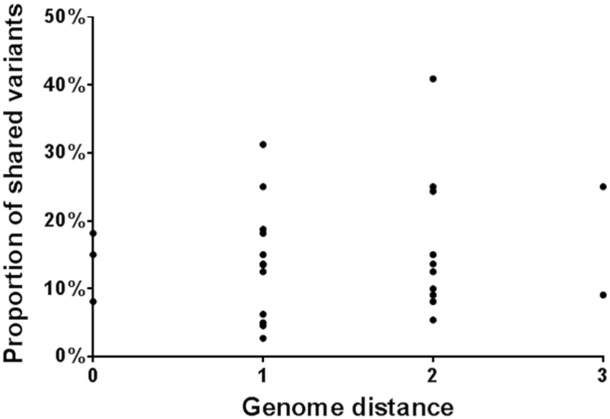
A pairwise comparison of the relationships between consensus sequences (genomic distance) and shared variants in the viral swarm populations. Each of the samples was compared to the other samples in turn, giving a total of 42 data points. Firstly the genome distance (number of nucleotide substitutions in the consensus sequences) between the samples was calculated. Secondly, the observed number of shared variants between the samples was divided by the total number of observed variants in one of the samples (as a consequence, a different value was generated when comparing samples X and Y than when comparing sample Y to X, to address differences in depth of coverage between the samples).

The hypothesis that as a virus disseminates through multiple hosts, the number of shared variants from the source of infection decreases, was investigated by a comparative analysis of each of the 7 samples to the other 6. No correlation between similarities in the swarm structure and the relatedness of consensus sequences was identified, suggesting that variation within a viral population is unique to the individual infected host (Fig. 4).

Finally viral swarm structures originating from different lesions in the same host were investigated further. Despite sharing identical consensus sequences and deriving from different lesions on the same animal, the swarm structures in sample 161A and sample 161B exhibited large differences in diversity. Twenty-two variants were identified in each lesion, however; only 4 were shared between both. Of the 4, two were fixed at consensus level with a greater than 99% frequency (VP2 – genome position 2429 and 3D – genome position 7428). The other two were located in the 5′UTR and VP1 and exhibited frequencies of 2.50% and 0.90% respectively (Fig. 3b). This is suggestive of distinct populations evolving independently at different site within the same host after dissemination and seeding from the initial infection site.

## 4 Discussion

This study describes the use of Illumina sequencing to dissect the consensus and sub-consensus dynamics of FMDV populations within samples collected from a sub-set of infected animals from a single cattle herd. Consensus sequencing is a powerful tool for reconstructing transmission networks between FMDV affected farms. In this study, the sequencing protocols utilised were validated, as sample 341 and sample 004 were previously characterised to consensus-level using Sanger sequencing (Valdazo-González et al., 2015), with both sets of sequences being identical (Fig. 2).

Statistical parsimony analysis was performed using the consensus sequences generated from each sample. The sequence of sample 341 with the oldest lesion age of 6 days (with sample 147) was most closely related to that of the consensus sequences generated from IP1b (Cottam et al., 2008) (Fig. 2), although caution should be exercised as we are unable to exclude other sources of transmission, nor the possibility that these viruses evolved independently within the animal to attain this level of shared genetic identity, independent of transmission. The results are also suggestive that sample 238 may act as an intermediary step prior to subsequent transmission to later IPs.

Previous studies have concluded that NGS can be used to examine the polymorphic diversity within a viral population at an unprecedented resolution to develop a better understanding of the fine-scale evolution of viruses (Wright et al., 2011, Fisher et al., 2015). A total of 109 consensus and sub-consensus changes were identified (Fig. 3), with the highest relative frequency occurring in the 2A encoding region (Table 2). Of these 109 variants, only 29 were shared between 2 or more samples and of this number, 1 variant was shared between 6 samples. The fact that a low number of variants were shared between 2 or more samples, of which the majority were either synonymous (54.54%) or located within the 5′UTR (9.09%) is possibly indicative of intra-herd viral evolution being largely neutral. Another explanation for this low shared variant frequency is a narrow transmission bottleneck event between infected animals, and as a result higher frequency substitutions are more likely to be transmitted. Although NGS technologies can be used to identify low-frequency mutations, the reconstruction of viral haplotypes remains problematic due to the short read length which often means that the distance between point mutations exceeds that of the read length (Schirmer et al., 2012), and process-introduced bias arising as a consequence of PCR amplification.

The results obtained here are consistent with the finding of previous studies that identified different swarm structures originating from separate lesions taken from a single infected host as part of an experimental transmission chain (Wright et al., 2011, Orton et al., 2013). In spite of sharing identical consensus sequences, both viral populations within samples 161A and 161B (i.e. samples deriving from the same host, but different lesion sites) exhibited different mutational profiles, with only 4 out of 22 variants above 0.5% in frequency shared between the two samples. This suggests that diversification of the viral swarm in these separate sites arises through independent stochastic mutation processes and in the absence of strong commonly shared positive selection pressures. As 2 of these variants were present at consensus level, it can be hypothesised that within a host, the transmission bottleneck is small and only the dominate virus populations disseminate between replication sites.

The large amount of diversity exhibited in the 7 samples coupled with the lack of shared variants may be indicative of viruses passing through evolutionary bottlenecks, with an expansion in viral swarm diversity arising as a consequence of local tissue replication. An alternative hypothesis is that mutational profiles are maintained through a process of reinfection between animals, maintaining variation within the viral swarm structures, as infected animals on a single IP are likely to repeatedly come in contact with one another. However due to the low number of variants shared between 2 or more infected animals, this is unlikely to be the case.

Pair-wise genomic changes identified within IP2b at a sub-consensus level were not correlated with consensus genomic different between pairs (Fig. 4). This appears to suggest that the generation of swarm structures arises similarly despite modest differences in the founding sequences, and further supporting the notion that diversification isn't subject to distinctive local selection pressures.

Deep-sequencing multiple samples taken from different host from within a single herd permitted the study of viral diversity on levels of both the individual host and that of the herd. The study has provided a novel, accurate and informative snapshot of viral diversity at a given time, which can have a potential impact on transmission dynamics. NGS technologies can provide a novel insight into the polymorphic frequencies that exist within a viral swarm population and can provide an understanding to the accumulation of viral diversity that occurs within a single IP during the course of a single outbreak.

## Figures and Tables

**Table 1:**
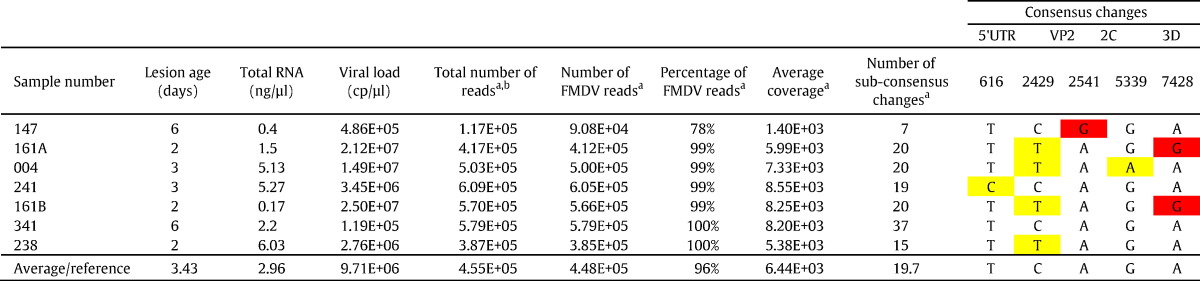
Details of estimated lesion age for each of the infected animals, with information regarding the RNA extraction and MiSeq sequencing. The sequence data presented represents an average for each of the duplicate samples. The table includes changes in consensus sequence compared to the reference sequence - EU448371, with synonymous changes indicated in yellow, non-synonymous changes indicated in red. The table also includes the number of sub-consensus changes present in each sample.

^a^Mean of two replicates. ^b^After sickle trimming.

**Table 2:** A list of total synonymous and non-synonymous changes present only at sub-consensus (below 50% frequency) level for each coding and UTR region for all samples sequenced.

Genome region	5′UTR	Leader	VP4	VP2	VP3	VP1	2A	2B	2C	3A	3B	3C	3D	3′UTR	Total^a^
Length of region	559	603	255	654	660	633	54	462	969	444	213	639	1410	86	
Synonymous	15	6	1	2	9	12	2	1	1	4	1	2	4	1	45
Non-synonymous		5	3	1	5	4	1	3	1	3	0	2	15		43
Total number of variants	15	11	4	3	14	16	3	4	2	7	1	4	19	1	
% (unique sites/region length)	2.68%	1.82%	1.57%	0.46%	2.12%	2.53%	5.55%	0.87%	0.21%	1.58%	0.47%	0.63%	1.35%	1.16%	

a Total number of synonymous or non-synonymous changes in the protein coding regions of the genome (excluding UTRs).

## Appendix A Supplementary data

Supplementary tables. Image 1
