# Supplementary material for: Investigating intra-host and intra-herd sequence diversity of foot-and-mouth disease virus
Source: Infect Genet Evol. 2016 Oct;44:286–92. doi: 10.1016/j.meegid.2016.07.010 (PMC5036933; doi:10.1016/j.meegid.2016.07.010)
Supplement: Supplementary file 1 — Supplementary tables. [file mmc1.docx]

| Genome Position | Sample 147 | Sample 161a | Sample 004 | Sample 241 | Sample 161b | Sample 341 | Sample 238 | Consensus base | Variant base | Genome region |
| --- | --- | --- | --- | --- | --- | --- | --- | --- | --- | --- |
| 616 |  |  |  | 46.309% |  |  | 37.197% | T | C | 5'UTR |
| 722 |  |  |  |  |  | 0.254% |  | C | T | 5'UTR |
| 727 |  |  |  |  | 0.994% |  |  | G | T | 5'UTR |
| 741 |  |  |  |  | 0.339% | 0.273% | 0.524% | C | T | 5'UTR |
| 748 |  |  |  |  |  | 0.303% |  | G | A | 5'UTR |
| 748 |  |  | 1.092% |  |  |  |  | G | T | 5'UTR |
| 818 |  |  |  |  | 0.379% |  |  | C | T | 5'UTR |
| 846 |  |  |  | 0.688% |  |  |  | C | T | 5'UTR |
| 864 | 4.252% |  |  |  |  |  |  | T | A | 5'UTR |
| 967 |  |  |  |  |  | 2.585% |  | G | A | 5'UTR |
| 1048 |  |  |  |  |  | 0.355% |  | G | A | 5'UTR |
| 1062 |  | 0.387% |  |  |  |  |  | A | G | 5'UTR |
| 1065 | 2.538% | 2.506% |  | 2.077% | 1.354% | 2.960% | 2.632% | A | G | 5'UTR |
| 1069 |  |  |  |  |  |  | 1.476% | C | T | 5'UTR |
| 1070 |  |  |  |  |  | 1.183% |  | T | G | 5'UTR |
| 1082 |  |  |  |  |  |  | 0.803% | T | A | 5'UTR |
| 1114 |  |  |  | 10.464% |  |  |  | C | T | Leader |
| 1127 |  |  |  |  | 2.318% |  |  | T | C | Leader |
| 1156 |  |  |  | 0.477% |  | 0.400% | 0.642% | G | A | Leader |
| 1197 |  |  |  |  | 0.427% |  |  | T | C | Leader |
| 1378 |  |  |  |  |  | 1.044% |  | C | T | Leader |
| 1442 |  |  |  | 0.278% |  |  |  | C | T | Leader |
| 1448 |  | 0.558% |  |  |  |  |  | G | A | Leader |
| 1484 |  |  | 0.463% |  |  |  |  | T | C | Leader |
| 1493 |  |  | 0.497% |  |  | 0.342% |  | A | G | Leader |
| 1568 |  |  |  |  |  |  | 3.419% | T | C | Leader |
| 1656 | 8.705% |  |  |  |  |  |  | G | A | Leader |
| 1702 |  |  | 0.605% |  |  |  |  | C | T | VP4 |
| 1801 |  |  |  |  |  | 0.733% |  | A | G | VP4 |
| 1803 |  |  |  |  |  | 0.679% |  | G | A | VP4 |
| 1910 |  |  |  |  |  | 0.439% |  | C | T | VP4 |
| 1972 |  |  |  |  | 0.455% |  | 0.785% | G | A | VP2 |
| 2330 |  |  | 1.127% |  |  |  |  | C | T | VP2 |
| 2429 | 12.181% | 99.426% | 99.838% |  | 98.690% |  | 60.085% | C | T | VP2 |
| 2474 |  |  | 0.464% |  |  |  |  | G | A | VP2 |
| 2541 | 89.204% |  |  |  |  |  |  | A | G | VP2 |
| 2811 |  |  |  |  |  | 0.458% |  | G | A | VP3 |
| 2833 |  |  | 0.274% |  |  |  |  | C | T | VP3 |
| 2862 |  |  |  |  |  | 0.433% |  | G | A | VP3 |
| 2900 |  |  |  | 0.268% |  |  |  | C | T | VP3 |
| 2933 |  | 1.082% |  |  |  |  |  | T | C | VP3 |
| 2945 |  |  |  | 10.342% |  |  |  | G | A | VP3 |
| 2959 |  |  |  |  | 1.079% |  |  | C | T | VP3 |
| 2960 |  | 1.167% |  |  |  | 0.534% |  | C | A | VP3 |
| 2963 |  | 0.916% |  |  |  |  |  | C | T | VP3 |
| 2984 |  | 0.663% |  |  |  | 0.495% |  | G | A | VP3 |
| 2987 |  | 1.206% |  |  |  | 0.517% |  | C | T | VP3 |
| 3008 |  | 1.794% |  |  |  |  |  | G | C | VP3 |
| 3013 |  |  |  | 0.297% |  |  |  | G | A | VP3 |
| 3263 |  |  | 10.568% |  |  |  |  | C | A | VP3 |

| Genome Position | Sample 147 | Sample 161a | Sample 004 | Sample 241 | Sample 161b | Sample 341 | Sample 238 | Consensus base | Variant base | Genome region |
| --- | --- | --- | --- | --- | --- | --- | --- | --- | --- | --- |
| 3271 |  |  | 0.567% |  |  |  |  | C | T | VP1 |
| 3296 |  | 1.909% |  |  |  | 0.751% |  | A | G | VP1 |
| 3308 |  |  |  |  |  | 0.637% |  | C | T | VP1 |
| 3311 |  |  |  |  |  | 0.988% |  | A | G | VP1 |
| 3318 |  | 2.552% |  |  |  | 1.217% |  | A | G | VP1 |
| 3353 |  | 1.674% |  |  |  |  | 0.563% | T | A | VP1 |
| 3380 |  | 0.938% | 0.470% |  | 0.366% | 1.808% |  | G | A | VP1 |
| 3397 |  |  | 0.724% |  | 0.858% |  |  | T | C | VP1 |
| 3420 |  |  |  |  | 0.627% |  |  | T | A | VP1 |
| 3563 |  | 1.979% |  |  |  |  |  | T | A | VP1 |
| 3644 |  |  |  |  |  |  | 10.039% | T | A | VP1 |
| 3650 |  |  | 0.396% |  |  |  |  | C | T | VP1 |
| 3728 |  | 2.276% |  |  |  | 1.041% |  | T | C | VP1 |
| 3770 |  | 1.497% |  |  |  |  |  | C | T | VP1 |
| 3773 |  | 1.368% |  |  |  |  |  | G | A | VP1 |
| 3902 |  | 4.071% |  |  |  | 1.035% |  | G | C | VP1 |
| 3909 |  |  |  | 0.503% |  |  |  | G | A | 2A |
| 3920 |  |  |  |  |  | 0.771% |  | T | G | 2A |
| 3962 |  | 2.291% |  |  |  |  |  | G | A | 2A |
| 4028 | 29.908% |  |  |  |  |  |  | T | C | 2B |
| 4177 |  |  | 1.102% |  |  |  |  | A | G | 2B |
| 4570 |  |  |  | 0.460% |  |  |  | A | G | 2B |
| 4647 |  |  |  | 0.415% |  |  |  | C | A | 2B |
| 4831 | 2.127% |  |  |  |  |  |  | C | T | 2C |
| 5099 |  |  | 0.577% |  |  |  |  | T | C | 2C |
| 5339 | 2.528% |  | 97.868% |  |  |  |  | G | A | 2C |
| 5525 |  |  |  |  | 2.927% |  |  | T | C | 3A |
| 5590 |  |  |  |  | 0.374% |  |  | G | A | 3A |
| 5597 |  |  |  |  | 1.419% |  |  | G | A | 3A |
| 5632 |  |  |  |  |  | 0.939% |  | T | C | 3A |
| 5715 |  |  |  | 0.423% | 0.328% |  |  | G | A | 3A |
| 5756 |  |  |  | 0.449% | 0.510% |  |  | C | T | 3A |
| 5795 |  |  | 3.551% |  |  |  |  | G | A | 3A |
| 5915 |  |  |  |  | 0.388% |  |  | G | A | 3B |
| 6119 |  |  |  |  | 0.428% |  |  | C | T | 3C |
| 6210 |  |  |  | 0.394% |  |  |  | A | G | 3C |
| 6244 |  |  |  |  |  |  | 0.915% | T | C | 3C |
| 6602 |  |  | 0.335% |  |  |  |  | C | T | 3C |
| 6712 |  |  |  |  |  |  | 0.569% | G | A | 3D |
| 6771 |  |  | 0.758% |  |  |  |  | G | A | 3D |
| 6934 |  |  |  |  |  | 0.333% | 0.542% | C | T | 3D |
| 6936 |  |  |  | 0.376% |  |  |  | G | A | 3D |
| 6980 |  |  |  |  |  | 0.319% | 0.494% | C | T | 3D |
| 7041 |  |  |  |  | 0.346% |  |  | C | T | 3D |
| 7198 |  |  |  |  | 0.332% |  |  | G | A | 3D |
| 7226 |  |  | 2.076% |  |  |  |  | T | C | 3D |
| 7289 |  |  | 0.332% | 0.338% |  |  |  | G | A | 3D |
| 7428 |  | 99.133% |  |  | 98.458% |  |  | A | G | 3D |
| 7474 |  |  |  |  |  | 0.334% |  | C | T | 3D |
| 7566 |  | 0.533% | 0.345% | 0.302% |  |  |  | G | A | 3D |
| 7614 |  |  |  |  |  | 0.265% |  | G | A | 3D |
| 7620 |  |  |  |  |  | 0.249% |  | C | T | 3D |
| 7626 |  |  |  |  |  |  | 3.183% | C | T | 3D |
| 7704 |  |  |  |  |  | 0.673% |  | T | C | 3D |
| 7730 |  |  |  | 3.537% |  |  |  | C | A | 3D |
| 7750 |  |  |  |  |  | 0.239% |  | C | A | 3D |
| 7871 |  |  |  | 0.476% |  | 0.444% |  | G | A | 3D |
| 8070 |  |  |  |  |  | 0.408% |  | G | A | 3D |
| 8125 |  |  |  |  |  | 14.837% |  | A | G | 3'UTR |

**Supplementary Table 1: A list of variants identified in both technical replicates for each sample**. This table shows the frequency of variants at given positions for each of the animals. The nucleotide change is given as well as the different genomic regions which are separated by a line. Only variants identified by Lofreq in both technical replicates were carried forward for investigation.

| **Sample**  **Name** | **No. of LoFreq Variants in Replicate 1** | **No. of LoFreq Variants in Replicate 2** | **No. of LoFreq Variants Shared**  **Between Replicates** | **% of LoFreq Variants Shared Between Replicates** |
| --- | --- | --- | --- | --- |
| 147 | 66 | 11 | 8 | 20.78% |
| 161a | 49 | 153 | 22 | 21.78% |
| 4 | 35 | 68 | 22 | 42.72% |
| 241 | 32 | 64 | 20 | 41.67% |
| 161b | 47 | 62 | 22 | 40.37% |
| 341 | 82 | 149 | 37 | 32.03% |
| 238 | 66 | 30 | 16 | 33.33% |

**Supplementary Table 2: Shared variants called by LoFreq between sample replicates.** This table shows the number of variants called by LoFreq in each of the two replicates for each of the 7 samples, along the number of variants that are called by LoFreq in both replicates. The percentages of shared variants between replicates is calculated as two times the number of shared variants between replicates, divided by the total number of variants in both replicates; two times as the shared variant is present twice – once in each replicate.
